# Sodium-Glucose Cotransporter-2 (SGLT-2) Inhibitors in Atrial Fibrillation: Clinical Implications, Mechanisms, and Therapeutic Potential

**DOI:** 10.7759/cureus.96689

**Published:** 2025-11-12

**Authors:** Mahmoud M Ramadan, Mohammad K Jawish, Omar Tamim, Mohamed T Abdelbary, Naya H Al-Refai, Sarah M Hady, Snds A Salama, Zahraa H Alhaboobi, Yasser A Abd-Alsamad, Hala H Alsabea, Mohammad T Allababidi, AbedAlhamid Alrais, Basmala H Zorek, Sara H Zorek, Mohamed A Saleh

**Affiliations:** 1 Department of Clinical Sciences, College of Medicine, University of Sharjah, Sharjah, ARE; 2 Cardiology, College of Medicine, Mansoura University, Mansoura, EGY; 3 Internal Medicine, Rashid Hospital, Dubai, ARE; 4 Medical School, College of Medicine, University of Sharjah, Sharjah, ARE; 5 Internal Medicine, Faculty of Medicine, Benha University, Benha, EGY

**Keywords:** atrial fibrillation, chronic kidney disease, diabetes, heart failure, sodium–glucose cotransporter-2 inhibitors

## Abstract

Atrial fibrillation (AF) is the most common sustained cardiac arrhythmia worldwide and contributes substantially to morbidity, mortality, and healthcare burden, particularly among patients with diabetes and heart failure (HF). Emerging evidence suggests that sodium-glucose cotransporter-2 (SGLT-2) inhibitors, initially developed as glucose-lowering agents, confer cardiovascular (CV) benefits that extend beyond glycaemic control. Recent clinical and experimental data indicate that these agents may reduce the incidence and burden of AF through multifactorial mechanisms involving hemodynamic, metabolic, and electrophysiological modulation. SGLT-2 inhibition improves myocardial energetics, enhances diastolic relaxation, and attenuates oxidative stress, inflammation, and atrial fibrosis - key determinants of atrial structural and electrical remodeling. In addition, these agents influence ionic homeostasis by modulating sodium and calcium handling, thereby stabilizing atrial conduction and preventing arrhythmogenic substrate formation. Observational analyses and post-hoc evaluations of major CV outcome trials, including DECLARE-TIMI and DAPA-HF, consistently show lower AF event rates among patients receiving SGLT-2 inhibitors. Preclinical models corroborate these findings, demonstrating reduced atrial remodeling and fibrosis independent of glycaemic status. Although current data are largely indirect and derived from secondary analyses, they collectively suggest that SGLT-2 inhibitors exert atrial-specific protective effects. Compared with traditional antiarrhythmic therapies, SGLT-2 inhibitors offer a complementary strategy that targets upstream metabolic and structural mechanisms rather than direct electrophysiological blockade. Ongoing studies aim to clarify their role in rhythm control, interaction with β-blockers, and efficacy in patients with pre-existing AF. While the safety profile remains favourable across age and comorbidity spectra, mechanistic uncertainties and the absence of dedicated randomized trials warrant further investigation. Understanding how SGLT-2 inhibition influences atrial remodeling could unveil a novel therapeutic avenue for the prevention and management of AF in both diabetic and non-diabetic populations.

## Introduction and background

The potential relationship between sodium-glucose cotransporter-2 (SGLT-2) inhibitors and atrial fibrillation (AF) arises from the diverse and pleiotropic mechanisms of these drugs beyond their well-established glycosuric action [1]. Experimental studies have associated AF with alterations in metabolism, ionic homeostasis, inflammation, and fibrosis. SGLT-2 inhibition - with its positive effects on energy metabolism and mitochondrial function, negative modulation of proinflammatory mediators, and reduction of fibrillar collagen deposition - could therefore modify atrial remodeling and the development of the arrhythmia [2]. Although these pathophysiological connections are not well established, results from several observational studies and a few interventional trials support a beneficial effect of SGLT-2 inhibitors on AF incidence, burden, or outcomes in subjects with heart disease and diabetes [3]. Cross-sectional or longitudinal analyses of large cardiovascular (CV) outcome trials have also yielded intriguing signals, suggesting that SGLT-2 inhibitors may be associated with reduced AF incidence or burden or improved rhythm-related outcome parameters, despite being neither antiarrhythmic agents per se nor specifically preferred for patients at higher AF risk [4].

The AF-specific effects of SGLT-2 inhibitors seem separate from their consistently reported CV protective properties; those derive from pleiotropic hemodynamic mechanisms, including effects on arterial stiffness, blood pressure, body weight, or heart failure (HF) risk, none of which directly relate to atrial remodeling [5]. Therefore, the specific links between SGLT-2 inhibition and atrial remodeling remain unclear, and existing research has not addressed this point yet. Such a summary would help identify compelling mechanistic connections, provide reassurance of drug safety and tolerability in patients with or at high risk of developing AF, and suggest avenues for further research [6]. Table 1 describes the mechanistic links between SGLT-2 inhibitors and atrial remodeling.

**Table 1:** Mechanistic Links Between SGLT-2 Inhibition and Atrial Remodeling Abbreviations: AMPK = AMP-activated protein kinase, IL = interleukin, NF-κB = nuclear factor-κB, ROS = reactive oxygen species, TNF-α = tumor necrosis factor-α, SGLT-2 = sodium-glucose cotransporter-2, eNOS = endothelial nitric oxide synthase, AF = atrial fibrillation, NLRP3 = nucleotide-binding domain, leucine-rich repeat, and pyrin domain-containing protein 3.

Mechanistic Domain	Cellular/Molecular Pathway	Experimental or Clinical Evidence	Expected Anti-AF Effect
Metabolic modulation	↑ AMPK, ↑ SIRT1 → improved mitochondrial efficiency, ↓ ROS	Dapagliflozin/empagliflozin animal models [7,8]	Restores atrial energetics, limits oxidative stress
Anti-inflammatory signaling	↓ NF-κB, ↓ NLRP3 inflammasome, ↓ IL-1β/IL-6/TNF-α	Cardiomyocyte & fibroblast cultures; diabetic rodents [7]	Reduces atrial cytokine burden and macrophage activation
Antifibrotic remodeling	↓ TGF-β1/Smad, ↓ α-SMA, ↓ collagen I/III	Dapagliflozin → less atrial fibrosis [8]	Improves structural integrity, conduction uniformity
Endothelial protection	↑ eNOS, ↓ ICAM-1/VCAM-1, ↓ Nox2	Endothelial-cell models [7]	Enhances nitric-oxide bioavailability, decreases atrial stiffness
Ion-channel modulation	↓ late INa, ↑ IKs, normalization of Ca2+ handling	Preclinical electrophysiology [1,3,5]	Stabilizes membrane potential, suppresses ectopy

Beyond glucose-lowering actions, SGLT-2 inhibitors have emerged as potent modulators of inflammatory pathways, with their cardioprotective effects increasingly linked to AMP-activated protein kinase (AMPK) activation and suppression of nuclear factor-κB (NF-κB) and NLRP3 inflammasome signaling. These anti-inflammatory actions may underlie their benefits in attenuating atrial remodeling and oxidative stress that predispose to AF [7].

Scientific evidence highlights that the SGLT-2 inhibitor dapagliflozin exerts direct cellular effects on cardiomyocytes, independent of glycaemic modulation. In vitro, dapagliflozin significantly attenuated isoproterenol-induced cardiomyocyte hypertrophy and oxidative stress by activating the phosphatidylinositol 3-kinase/protein kinase B (PI3K/AKT) pathway and suppressing inflammatory mediators such as NLRP3 and tumor necrosis factor-α (TNF-α), while restoring endothelial nitric oxide synthase (eNOS) expression and reducing reactive oxygen species (ROS) generation [8].

## Review

Rationale for studying SGLT-2 inhibitors in atrial fibrillation

The incidence of AF continues to grow as the population ages and the prevalence of related comorbidities increases. AF significantly raises the risk of stroke, HF, and other CV events, leading to a greater burden of mortality, morbidity, and hospitalization [9]. Consequently, it is imperative to develop effective therapeutic strategies to address this public health challenge. Dapagliflozin, the SGLT-2 inhibitor approved for clinical use, was studied in the Dapagliflozin and Cardiovascular and Renal Outcomes in Patients with Heart Failure and Diabetes or Pre-diabetes (DAPA-HF) trial, which included patients with HF and preserved or mildly reduced ejection fraction [10].

Dapagliflozin was shown to attenuate atrial remodeling and delay the onset of AF in animal models, while in the DAPA-HF trial itself, dapagliflozin was associated with reduced AF burden and improved heart rate control [11]. Moreover, post-hoc analysis from the Dapagliflozin and Cardiovascular and Renal Outcomes in Patients with Heart Failure and Diabetes or Pre-diabetes (DECLARE-TIMI-58) trial suggested that dapagliflozin may decrease the risk of new-onset AF in patients with type 2 diabetes who have established CV disease or are at high CV risk [12,13]. SGLT-2 inhibitors should therefore be considered potential therapeutic options against incident and recurrent AF.

Overview of atrial fibrillation pathophysiology

AF is the most prevalent sustained arrhythmia. Global estimates show that, in 2019, seven million people had AF; this number is projected to increase to 14-16 million by 2060 [12]. AF doubles all-cause mortality [14] and markedly increases the risks of heart failure and dementia [9]. Moreover, AF elevates the risk of stroke by approximately four- to fivefold and of cognitive impairment by about threefold [9]. Diabetic patients have approximately 40% higher odds of developing AF [14]. Despite effective management of conventional risk factors, the prevalence of AF continues to rise steeply. Available treatment options, including antiarrhythmic drugs and invasive procedures, carry significant risks.

AF is a common and undertreated condition in patients with type 2 diabetes. SGLT-2 inhibitors, which are used to treat type 2 diabetes, have been shown to reduce the incidence of AF [6]. SGLT-2, a sodium-glucose cotransporter, plays a central role in glucose reabsorption in the kidneys and represents the primary target for glucose-lowering treatments [15].

Pharmacology of SGLT-2 inhibitors

SGLT-2 inhibitors are a novel class of antihyperglycemic drugs that inhibit glucose and sodium reabsorption in the proximal renal tubule, thereby lowering blood glucose and increasing urine output [12]. Their initial development targeted diabetes, but other effects have emerged. SGLT2 inhibition reduces renal hyperfiltration and sympathetic nerve activity, while promoting diuresis, natriuresis, glucose excretion, and a modest reduction in blood pressure; all of which help to reduce cardiac workload [5]. Myocardial SGLT-2 expression is negligible in humans. SGLT-2 inhibitors act primarily in the kidney, lowering imported sodium and altering intracellular cation and glucose homeostasis [12].

These cellular findings complement the hemodynamic benefits by revealing a molecular axis-PI3K/AKT-NRF2 signaling-that counteracts oxidative and inflammatory injury [8]. Dapagliflozin downregulated pro-fibrotic pSMAD and α-SMA expression and normalized NHE-1 and SGLT-2 activity, thereby improving intracellular ion balance [8].

SGLT-2-inhibitor pharmacokinetics and pharmacodynamics are known for FDA-approved agents; concentrations decline logarithmically after one to two hours [16]. Routes include oral, with half-lives of five to 13 hours and binding to plasma proteins of 75-97% [16]. CV safety signals emerged in phase 3 trials of canagliflozin and dapagliflozin as shares of major adverse CV events, hospitalizations for HF, and CV deaths declined. The FDA recommended long-term outcome trials which revealed favourable outcomes and results for SGLT-2 inhibition [16,17].

Mechanisms of Action Beyond Glucose Lowering

SGLT-2 inhibitors are a new class of antidiabetic agents that lower blood glucose independently of insulin action. Current evidence suggests a range of types 1 and 2 diabetes and non-diabetic effects, including CV and renal protection [12]. A proposed mechanism for SGLT-2 inhibitors is enhanced natriuresis, which has been linked to AF. SGLT-2 inhibitors have an impact on several organ systems, including the cardiac myocyte, which may alter the progression towards AF [18]. Dapagliflozin decreased the incidence of AF compared with placebo in patients with established CV disease and/or moderate chronic kidney disease (CKD) receiving background therapy [19]. Only dapagliflozin has been studied in randomized trials. Comparative agents gliflozins have a similar CV safety profile [13].

At the molecular level, SGLT-2 inhibition activates the AMPK/SIRT1 axis, promoting autophagy and reducing oxidative stress in cardiomyocytes and endothelial cells. Inhibition of TLR-4 expression and NF-κB phosphorylation further suppresses inflammatory cytokines such as IL-6, IL-1β, and TNF-α, thereby improving cellular redox homeostasis and mitochondrial integrity. These effects converge on preserving atrial electrophysiologic stability through reduced ion channel dysregulation and structural fibrosis [7].

Endothelial dysfunction, a hallmark of atrial remodeling, is alleviated by SGLT-2 inhibitors through enhanced nitric oxide bioavailability and downregulation of NADPH oxidase (Nox2) expression. Empagliflozin and dapagliflozin have been shown to restore endothelial AMPK activity and reduce adhesion molecule expression (ICAM-1, VCAM-1), thus improving atrial endothelial resilience against pro-arrhythmic inflammation [7].

Pharmacokinetics and Pharmacodynamics

Dapagliflozin inhibits SGLT-2 without affecting the related transporter SGLT-1 [16,17]. Although SGLT-2 inhibitors primarily aim to reduce hyperglycemia in patients with type 2 diabetes, they exert additional effects that may contribute to the observed reduction of CV events, hospitalization for HF, and overall mortality. Observational studies of dapagliflozin and empagliflozin have associated the use of SGLT-2 inhibitors with reduction in AF incidence and burden in diabetic patients [20,21].

Cardiovascular Safety and Prior Outcomes in Other Diseases

SGLT-2 inhibitors were originally developed for glycemic regulation in type 2 diabetes mellitus. However, CV, renal, and mortality benefits in patients with established atherosclerotic CV disease and/or HF led to their approval for broader use. Guidance on prescribing decisions still relies on specific indications [4]. At the same time, emerging evidence suggests that SGLT-2 inhibition may also exert influence over cardiac arrhythmias. In particular, substantial observational and real-world data document significant, albeit indirect, signals about the modulatory effects of SGLT-2 inhibitors on AF, one of the most common arrhythmias associated with a high burden of strokes and thromboembolism [18,22]. Systematic reviews of large meta-analyzes show that both incidence and burden of AF in patients with established CV disease, HF, CKD, or diabetes - and patients without prior AF - are consistently reduced [23].

While direct clinical trials remain to conclusively define whether SGLT-2 inhibitors may constitute a valid therapeutic adjunct to standard rhythm control therapies indicated for patients with nonvalvular AF, and the underlying mechanism seem to support this. Experimental preparations revealed a necessarily broader spectrum of interrelated actions, with consequences for AF miles, risk determinants, and therapeutic options [24]. Clinical indications for frontline use are consistently noted, with a sharply decreased risk of serious AF-related events. These detailed findings could accommodate diverse contemporary roles of SGLT-2 inhibitors in initial rhythm control interventions. Consequently, prior analysis of CV safety and associated outcomes across diverse other indications assumes additional relevance, both to timely frame population pharmacovigilance policies and to flesh out complementary approaches to local real-world considerations [13,25-27].

Pathophysiological links between SGLT-2 inhibition and atrial remodeling

Comorbidities associated with diabetes and HF, such as AF, contribute substantially to morbidity and mortality. SGLT-2 inhibitors have emerged as an effective treatment for patients with HF with reduced ejection fraction (HFrEF) of any etiology [10]. Clinical studies show a reduction in AF incidence and burden with SGLT-2 inhibition, potentially mediated by atrial structural and electrical remodeling [13,28]. AF initiation, maintenance, and progression result from complex interrelated changes in atrial substrate and electrophysiology [15]. SGLT-2 inhibitors, alone or in combination with traditional anti-arrhythmics, show complementary mechanisms and effects on metabolic and structural risk factors [29]. SGLT-2 inhibition decreases weight and fat mass, with reduction in body and epicardial fat, and lowers inflammation, fibrosis, ventricular mass, hypertrophy, and pressure, all of which reduce AF risk [30]. SGLT-2 inhibitors counter dysregulated myocardial energetics and mitochondrial dysfunction in HF, improving diastolic calcium handling and function [31,32].

SGLT-2 inhibitors modulate multiple ion channels implicated in AF. Reduced sodium currents (INa) and increased I_K inhibition decrease AF vulnerability, while reduced late INa and enhanced I_Ks restoration help prevent tachyarrhythmias and restore diastolic potentials [33-36].

The atria play a central role in AF pathophysiology, being the site for both AF initiation and maintenance. An anatomically and electrically heterogeneous substrate is important in the continuum of AF, including one of its main drivers: ectopic activity. Microstructural heterogeneity of the atrial conduction system is increased. Structural abnormalities are encountered with higher frequency in persistent AF, a predominance of connective and inflammatory tissue at the front stage of the disease [32,37,38]. The observed activation of the PI3K/AKT pathway by dapagliflozin also aligns with reduced atrial fibrosis and remodeling pathways implicated in AF. Upregulation of NRF2 and antioxidant genes (HO-1, NQO1) suggests attenuation of oxidative injury - a pivotal driver of atrial structural remodeling [8].

Ion Channel Modulation and Electrophysiological Effects

AF remains one of the most important risk factors for CV morbidity and mortality [39,40]. Patients suffering from type 2 diabetes have a 50-100% increased AF incidence when compared to non-diabetics. Older patients and those with established coronary artery disease (CAD) are labelled at higher risk for type 2 diabetes and associated complications such as AF [41]. SGLT-2 inhibitors have been proposed as a treatment option because they go beyond glucose lowering. Multiple clinical studies have shown that SGLT-2 inhibitors (canagliflozin, empagliflozin, dapagliflozin, and ertugliflozin) can significantly improve antiarrhythmic actions [24,33].

Preclinical studies provide evidence of the pathophysiological links between SGLT-2 inhibition and atrial remodeling. SGLT-2 inhibitors regulate ion channels involved in determining cardiac excitability, propagation, and contractility. Ion channel modulation may therefore affect recovery times during the cardiac cycle [1,42,43]. Evidence has emerged that SGLT-2 inhibition improves myocardial energetics, and diastolic dysfunction is also involved in the pathophysiology of AF [1,44]. The increase in AF burden estimated the effect in one clinical study for dapagliflozin compared to a baseline period without SGLT-2 inhibitors. AF burden decreased and no other CV endpoint was affected, strongly suggesting a potential mechanistic link [15].

Myocardial Energy Metabolism and Diastolic Function

Impairment of myocardial energy metabolism has been linked to diastolic dysfunction; both of these phenomena drive atrial remodeling [5,45]. Increased fibrotic and collagen deposition, together with hypertrophy and inflammation, induce defects in intracellular calcium handling, impairing the normal calcium transient decrease during diastole [44]. The consequent prolongation of calcium transients leads to defects in diastolic relaxation and elevated diastolic pressure [45]. Detrimental alterations of metabolic homeostasis occur at the earliest stage of atrial hypertrophy and persist in AF [46]. The heart relies primarily on glucose and fatty acid oxidation (FAO) pathways to generate energy. All major metabolic pathways, including glycolysis, ketogenesis, ketolysis, FAO, tricarboxylic acid cycle, and oxidative phosphorylation, are activated to sustain ATP levels at the sole level necessary to maintain atrial electromechanical function after atrial overload [1,47].

All these metabolic alterations are consistent with an even higher demand for ATP during AF than during sinus rhythm. As the AF progresses, these ATP-maintaining mechanisms shift to an “excess ATP supply” state. The increased dependency on fatty acid metabolism, although efficient, is associated with an excessive generation of ROS [48]. An excessive production of superoxide anions occurs during the first few minutes of AF in both animal models and patients, leading to diastolic intracellular calcium overload and induction of electrical remodeling [49].

Inflammation, Fibrosis, and Atrial Remodeling

AF development and progression are also closely linked to inflammation and fibrosis, which promote atrial remodeling through several mechanisms. In particular, inflammatory cytokines released in response to hypertrophy or ischemia modulate cardiac ion channels, altering Na+, Ca2+, and K+ conductance [12]. TNF-α, the cytokine most commonly associated with AF, induces atrial structural remodeling through pathways involving nuclear factor-κB, extracellular-signal-regulated kinase 1/2, and protein kinase D [46]. Dapagliflozin reduced inflammatory cytokines IL-1β, IL-6, and TNF-α in vitro and mitigated endoplasmic reticulum stress marker GRP78, demonstrating a potent anti-inflammatory and anti-fibrotic profile. Such cellular modulation parallels the reduction in atrial fibrotic burden observed in experimental models of AF [8].

Chronic atrial inflammation contributes to structural remodeling and arrhythmogenesis. Preclinical models demonstrate that SGLT-2 inhibitors, particularly dapagliflozin and empagliflozin, attenuate myocardial NLRP3 activation and cytokine release, leading to reduced collagen deposition and fibrosis. Additionally, these agents modulate macrophage polarization from pro-inflammatory M1 to reparative M2 phenotypes, a shift that mitigates atrial tissue inflammation and supports electrical stability [7].

Both experimental and translational data highlight SGLT-2 inhibitors’ ability to inhibit TGF-β1/Smad and ROS-NLRP3-caspase-1 pathways, leading to suppression of fibroblast-to-myofibroblast transformation and reduction in extracellular matrix accumulation. In diabetic and non-diabetic models, empagliflozin and dapagliflozin significantly reduced cardiac collagen synthesis and improved left atrial compliance [7].

Subepicardial fibrosis, which is both atrial-specific and determined during the first three days following myocardial infarction, can develop in the atria during the neonatal period. Transgenic expression of redox-sensitive green fluorescent protein under the control of the atrial-specific myosin light chain-2 promoter reveals that atrial myocyte redox status is more oxidized than that of ventricular myocytes in healthy hearts and is maintained throughout the life span in both cardiac regions [50]. During the progression of heart disease, however, atrial myocyte redox status becomes the most reduced among all regions, contrasting with the more oxidized levels observed in the ventricle. As a result, the earliest redox depolarization events, which signify the initiation of second harmonic bursting, start from the atria and drive the conduction pattern across the entire heart. Hence, maintaining atrial redox status appears to be a promising target for preventing AF [51-53].

Diastolic dysfunction related to increased ionic imbalances and perturbations in calcium homeostasis also forms a pivotal component of AF pathophysiology. Elevated left atrial pressure, a consequence of diastolic dysfunction, increases pulmonary vein volume and dilatation, which are predictive of AF [54-56]. Furthermore, electrical coupling between the pulmonary veins and the left atrium may strengthen as pulmonary vein smooth muscle cells undergo hypertrophy/dilated remodeling, augmenting AF vulnerability [57,58]. Maintaining diastolic function through SGLT-2 inhibition could prevent or delay these downstream changes, thereby exerting an upstream effect on AF [59].

Clinical evidence in atrial fibrillation

SGLT-2 inhibitors may influence new-onset AF, AF burden, or CV endpoints in high-risk populations with diabetes and at least one established CV risk factor [12]. Post-hoc analyses of the DECLARE-TIMI 58 trial suggested a significantly lower incidence of AF among dapagliflozin-treated patients [60]. SGLT-2 inhibition remains a candidate for upfront pharmacotherapy to ameliorate pre-existing AF on multiple risk factors. Dapagliflozin inhibited the incidence of new AF or flutters, as well as composite CV outcomes in high-risk, CV-proven diabetes patients [13,61]. In patients receiving β-blockers, the additional dapagliflozin quantum still returned a lower burden of AF observed through both new-onset intervals and total number of flutters [13]. Dapagliflozin also subsequently lowers composite CV outcomes among patients with pre-existing flutters when used either alone or with β-blockers [10,13]. These clinical observations are mechanistically supported by experimental data showing that dapagliflozin reverses isoproterenol (ISO)-induced hypertrophy and oxidative stress through PI3K/AKT activation and NHE-1 downregulation, linking cellular cardio-protection with clinical rhythm benefits [8]. Table 2 summarizes the evidence from major trials and analyses on AF outcomes with SGLT-2 inhibitors.

**Table 2:** Evidence from Major Trials and Analyses on AF Outcomes With SGLT-2 Inhibitors Abbreviations: AF = atrial fibrillation, ASCVD = atherosclerotic cardiovascular disease, CKD = chronic kidney disease, CV = cardiovascular, HF = heart failure, HFpEF = heart failure with preserved ejection fraction, HFrEF = heart failure with reduced ejection fraction, MACE = major adverse cardiovascular events, T2DM = type 2 diabetes mellitus, SGLT-2 = sodium-glucose cotransporter-2.

Trial / Study	Population	Agent	Primary CV Outcome	AF-Specific Finding	Reference
DECLARE-TIMI 58	T2DM ± ASCVD	Dapagliflozin 10 mg daily	↓ MACE trend	↓ new-onset AF by ~19 %	[12,13,60]
DAPA-HF	HFrEF ± T2DM	Dapagliflozin 10 mg	↓ CV death / HF hospitalization	↓ AF burden, improved rate control	[10,11]
EMPA-REG OUTCOME	T2DM + ASCVD	Empagliflozin 10–25 mg	↓ CV mortality	Trend to ↓ AF events (not powered)	[62]
CANVAS / CREDENCE	T2DM + CKD	Canagliflozin 100–300 mg	↓ HF hosp.	Neutral for AF	[62,63]
Meta-analyses (2020-24)	Pooled CV/renal trials	Mixed	—	15–25 % ↓ incident AF vs control	[23,26,27]

Observational Studies and Real-World Data

The first randomized trials to evaluate the CV safety of SGLT-2 inhibitors demonstrated signals of reduced HF hospitalization and CV death [62]. Secondary analyses of the CANVAS and DECLARE trials provided consistent evidence that these agents are associated with a lower incidence of AF [61,62]. The EMPAREG trial suggested a numerically smaller effect on HF-related outcomes, while CANVAS and CREDENCE reported no significant association of the agents with this arrhythmia [62,63].

In large, randomized trials targeting metabolic and renal risk factors, SGLT-2 inhibitors demonstrated additive value within established cardioprotective regimens, including angiotensin-converting enzyme (ACE) inhibitors, angiotensin receptor blockers (ARBs), and mineralocorticoid-receptor antagonists (MRAs). Observational and real-world studies report an upward trend in glucose-lowering prescriptions, specifically SGLT-2 inhibitors combined with other anti-diabetic agents, among patients with AF; hypothesis-generating analyses indicate these agents may target the arrhythmic substrate itself, rather than solely influencing CV or metabolic risk factors [25]. SGLT-2 inhibitors appear to reduce the incidence of serious AF events in patients with HF [4] or diabetes [60].

Randomized Trials Relevant to AF Outcomes

Atrioventricular estimates of AF incidence according to the type of SGLT-2 inhibitor and trial population revealed no significant differences [60]. No signal attributable to SGLT-2 inhibitor was observed for nonspecific electrocardiographic events or for other CV longitudinal end points investigated in parallel to AF. Randomized trials evaluated SGLT-2 inhibitors in HFrEF and metabolic syndrome, as well as CV sequelae among participants with type 2 diabetes [10,64]. AF counts were documented through reports of serious adverse events warranting hospitalization, for which incident AF should represent a well-established clinical endpoint. Observational studies show SGLT-2 inhibitor versatility: major anuria, incidental ketoacidosis, or composite safety effects on the CV apparatus are rarely encountered and, when present, seldom prompt admission [4].

Comparative and combined therapies

Both SGLT-2 inhibitors and traditional antiarrhythmic drugs are thought to influence the same distal electrophysiological substrate and reverse atrial remodeling. Prior studies link SGLT-2 inhibitors to lower rates of new-onset or recurrent AF compared with patients on metformin or other glucose-lowering monotherapies [15]. Metformin, though not devoid of effects on ionic homeostasis, has relatively weaker impacts on AF-predisposing factors. In a matched cohort analysis of >12,000 patients initiating empagliflozin or metformin in a real-world setting, SGLT-2 inhibition conferred a 25% lower hazard for AF diagnosis [65].

Trials randomizing SGLT-2 inhibitor versus placebo add weight to the association, revealing significant reductions in AF-related endpoints across the spectrum of CV disease and metabolic dysfunction [60]. Combined with findings in diverse preclinical models (infarction, pressure overload, diabetes, obesity, and aging with parallel mitigation of AF triggers, substrate, and electric remodeling [60]), the preemptive designation as a protective agent is warranted. SGLT-2 inhibitors might synergize with β-blockers to reinforce heart-rate control in a rhythm-control strategy [1]. Inhibition of SGLT-2 reduces glucose-driven activation of the renin-angiotensin-aldosterone system (RAAS), producing a diuretic effect similar to loop diuretics but without excessive potassium loss [66]. Unlike hyperglycemia-associated sodium channel activation that favors arrhythmogenesis, SGLT-2 inhibition leads to favorable ionic balance and sustained antiarrhythmic benefits in AF [67]. Optimizing the timing of adjunctive therapies, such as β-blockers, metformin, or dipeptidyl peptidase-4 (DPP-4) inhibitors, may further reduce AF recurrence, and dual therapy could represent an alternative strategy to conventional sequential administration [68,69].

SGLT-2 Inhibitors Versus Traditional Antiarrhythmics

The antiarrhythmic potential of SGLT-2 inhibitors has gained increasing attention. Their profile contrasts sharply with traditional antiarrhythmics, which predominantly focus on ion channel interactions and electrical properties. Available evidence suggests that SGLT-2 inhibitors, although devoid of dedicated antiarrhythmic trials, exhibit effects on AF not seen with other CV treatments [15]. Mechanisms linking SGLT-2 inhibition to atrial remodeling diverge from classical paradigms, yet several of these mechanisms exert direct electro-mechanical effects on atrial tissue relevant to arrhythmia initiation and maintenance [1,18,29]. In parallel, SGLT-2 inhibitors modulate CV systems that indirectly impose remodeling on atrial substrate. Much of the literature detailing such mechanisms stems from HF models. A more comprehensive understanding of the underlying processes is needed to reconcile the observed discrepancies between their direct and indirect effects [60]. Table 3 summarizes the comparative mechanistic spectrum between SGLT-2 inhibitors and traditional antiarrhythmics.

**Table 3:** Comparative Mechanistic Spectrum: SGLT-2 Inhibitors vs Traditional Antiarrhythmics Abbreviations: AMPK = adenosine monophosphate–activated protein kinase, ATP = adenosine triphosphate, CV = cardiovascular, HF = heart failure, HF hosp. = heart failure hospitalization, NLRP3 = nucleotide-binding domain, leucine-rich repeat, and pyrin domain-containing protein 3, ROS = reactive oxygen species, TGF-β = transforming growth factor beta, SGLT-2 = sodium-glucose cotransporter-2.

Mechanistic Target	Class I/III Antiarrhythmics	SGLT-2 Inhibitors	Clinical Implication
Ion-channel blockade	Direct Na⁺/K⁺ block	Indirect modulation via AMPK, Ca²⁺ handling	Fewer pro-arrhythmic risks
Metabolic regulation	None	↑ AMPK → ↑ ATP, ↓ ROS	Supports energetic stability
Fibrosis / inflammation	Minimal	↓ TGF-β, ↓ NLRP3	Structural reverse remodeling
Hemodynamic effect	Neutral or negative in HF	↓ preload & afterload (through natriuresis)	Improved diastolic function
Long-term mortality	Neutral	↓ HF hospitalization & CV death	Prognostic advantage

Synergy With Beta-Blockers and Rhythm Control Strategies

SGLT-2 inhibitors exert antiarrhythmic effects distinct from traditional Class I or III agents, as they target diastolic mechanical alternans rather than atrial ectopic foci [1,60]. The completion of formal rhythm-control trials with traditional antiarrhythmic agents in populations also treated with SGLT-2 inhibitors creates an opportunity to explore potential synergy. β-blockers, frequently selected when rhythm-control strategies are employed, similarly attenuate diastolic alternans and appear to act through different mechanistic pathways [70]. SGLT-2 inhibitors are hypothesized to enhance these effects, and careful consideration of existing evidence and physiopathological interactions across the SGLT-2 pharmacology, AF, and HF sections supports prioritizing rhythm-control strategies when initiating SGLT-2 treatment [60,71]. The fundamental pathways linking SGLT-2 inhibition to dromotropic and inotropic modulation render temporal sequencing unimportant; initiation of glucose-lowering therapy at any time point after rhythm-control engagement remains sensible [60].

Safety, tolerability, and special populations

AF is frequently diagnosed among diabetic patients; the electrocardiogram of AF patients often shows indistinctness of P-wave. Nevertheless, significant trials and animal studies have demonstrated the effectiveness of SGLT-2 inhibitors in treating HF in diabetic and non-diabetic patients. SGLT-2 inhibitors indirectly play such a role in AF patients by preventing the aggravation of HF [72]. The SGLT gene has two subtypes, SGLT-1 and SGLT-2, both of which, when knocked down, may lead to atrial enlargement [1]. SGLT-1 and SGLT-2 work synergistically to help maintain cellular electrolyte homeostasis. Activation of SGLT pathways can stimulate guanylate cyclase activity, which in turn promotes the opening of voltage-gated calcium channels (particularly Cav2.2), thereby preserving intracellular calcium concentrations within the physiological range [73].

SGLT-2 inhibitors have the potential to serve as a novel line of treatment in managing AF. Class and type of SGLT-2 inhibitors may affect the extent of AF mitigation; canagliflozin and empagliflozin have been reported to be more effective in the reduction of AF and atrial stretch from animal studies. They can also facilitate the activity of anti-AF agents, for instance, dronedarone, ivabradine, and metoprolol [60,74].

Renal Function Considerations

A decline in renal function is common in patients with HF, AF, or diabetes. Despite the increased risk of deterioration in renal function associated with diuretics and the approved CV protective medications, a significant percentage of patients have persistent volume overload because of insufficient volume control [75]. SGLT-2 inhibitors reduce atrial structural and electrical remodeling, including left atrial diameter and fibrosis, partly through inhibiting PGC-1a/NRF-1/Tfam pathways; and treatment with other SGLT-2 inhibitors has improved measures like atrial size, calcium handling, and epicardial fat volume that are linked to AF [76].

Age, Comorbidities, and Gender Differences

The influence of SGLT-2 inhibitors on the incidence and burden of AF appears to be consistent in various subpopulations defined by age, comorbidity, and female gender. Age and comorbidity directly impact tolerance and adherence to medications in individuals with diabetes, which represents an important factor in the prescription of drugs. SGLT-2 inhibitor-related adverse events that can complicate their use include transient mild dehydration, urinary tract infections, genital mycotic infections, and rarely euglycemic diabetic ketoacidosis (DKA) [77].

Practical implications for clinicians

The effects of SGLT-2 inhibition on heart rhythm are attracting growing interest in view of the emerging clinical signals from several large CV outcomes trials [12]. Early observations suggested that combined use of SGLT-2 inhibitors and DPP-4 inhibitors - a regimen that directly targets atrial metabolic derangements - might achieve more efficient risk reduction than either agent alone, although these analyses did not establish causal links between dual peroxisome proliferator-activated receptor (PPAR) agonist and arrhythmia and did not account for partial cotreatment with DPP-4 inhibitors [78].

Randomized trial data have consistently associated SGLT-2 inhibitors with a significantly lower incidence and burden of new-onset AF, although the precise mechanisms underlying these signals remain uncertain [15]. In particular, the atrial-specific electrical and structural changes mediating effective rhythm control in the type 2 diabetic setting lack experimental confirmation. The relationship between major adverse cardiovascular events (MACE) and AF remains poorly defined. Distinguishing the direct mechanistic effect of SGLT-2 inhibitors from metabolic area treatment therefore occupies an important position in contemporary knowledge translation [13].

Guidance on patient identification, stratification, and combination with rhythm-control strategies therefore occupies an important position in contemporary knowledge translation. Observational data indicate that clinical signals appear strongest and most consistent among patients with established AF and additional relevant comorbidities. In AF populations, safety profiles and frequencies of adverse events with SGLT-2 inhibition differ relative to a non-AF cohort. Clinicians assessing SGLT-2 inhibitors in patients undergoing rhythm-control interventions face complementary but also distinct considerations compared to those selecting agents for other populations [60]. Table 4 shows the clinical and practical implications for AF management.

**Table 4:** Clinical and Practical Implications for AF Management Abbreviations: AF = atrial fibrillation, CKD = chronic kidney disease, DKA = diabetic ketoacidosis, HF = heart failure, RAAS = renin–angiotensin–aldosterone system, T2DM = type 2 diabetes mellitus, UTI = urinary tract infection, SGLT-2 = sodium-glucose cotransporter-2.

Clinical Aspect	Implication of SGLT-2 Inhibitor Use	Supporting Evidence
Patient selection	Greatest benefit in T2DM + HF or CKD with AF risk	DAPA-HF post-hoc [11], DECLARE [13]
Therapeutic timing	Safe to initiate concurrently with rhythm control	[60,71]
Combinational strategy	Synergistic with β-blockers and RAAS blockers	[70–72]
Safety profile	Mild UTI/genital infection; rare DKA	[77]
Monitoring	Renal function and volume status periodically	[75]

Patient Selection and Risk Stratification

A broad spectrum of data collected from observational studies, real-world evidence, and post-hoc randomized clinical trials indicate that SGLT-2 inhibitors reduce the incidence and burden of AF. Results demonstrate a significant decrease in AF events associated with long-term SGLT-2 inhibition, with a clinically relevant signal consistently detected across major CV outcome trials. In contrast, non-AF CV endpoints did not show a statistically significant difference for AF. These findings suggest that SGLT-2 inhibition may have an atrial-specific effect linked to the pathophysiological mechanisms that underlie the metabolic syndromes and CV disease commonly associated with AF [12].

Dosing Strategies and Monitoring for AF Patients

Current AF management strategies are not satisfying, which leads to search for additional therapies that may improve the disease. Dosing and administration of SGLT-2 inhibitors rely on other comorbidities and individual patient characteristics [60]. SGLT-2 inhibitors can be initiated at standard doses. In the absence of other specific indications for dosage adjustment, metformin is the safest and most commonly used concomitant medication in patients with DM and AF [62]. Testing other pharmacological combinations remains essential in the search for additional therapies that may improve outcomes.

SGLT-2 inhibitors reduce glucose levels without increasing the risk of hypoglycemia. Clinical practice guidelines recommend prioritizing SGLT-2 inhibitors in patients with CKD or HF. The beneficial effects of SGLT-2 inhibitor medications in patients with type 2 diabetes have been shown to significantly reduce the rate of hospitalization for HF and CV death [4]. Further clinical studies are needed to determine the optimal clinical setting for prescriber initiation of approved SGLT-2 inhibitor medications.

Mechanistic gaps and future research

A variety of mechanistic questions remain unresolved regarding the effects of SGLT-2 inhibitors on atrial physiology that could further refine clinical hypotheses and experimental plans. These drugs mitigate AF risk despite SGLT-2 expression being limited to the renal proximal tubule [15]. Atrial-specific targets, potentially modulatory of conduction or repolarization, have not been identified, nor has an exhaustive list of candidate pathway-plasma biomarker relationships [12].

The absence of standardized definitions and consistent quantification methods for structural, electrical, and metabolic aspects of atrial remodeling hampers the ability to identify which specific domains are responsible for the observed clinical benefits. Insight into the sequence of atrial structural alteration, diastolic dysfunction, and AF development would establish a temporal framework to inform cross-sectional analyses [79,80]. Finally, determining how atrial adaptation modulates the relationship of other reported SGLT-2 effects to AF onset or progression would be valuable for prioritizing between competing mechanistic proposals [12,67]. Table 5 shows the remaining mechanistic gaps and future research priorities.

**Table 5:** Remaining Mechanistic Gaps and Future Research Priorities Abbreviations: AF = atrial fibrillation, DPP-4 = dipeptidyl peptidase-4, HF = heart failure, MRI = magnetic resonance imaging, RCTs = randomized controlled trials, SGLT-2 = sodium–glucose cotransporter-2.

Knowledge Gap	Current Limitation	Proposed Research Direction
Atrial-specific SGLT-2 expression	Expression mainly renal	Transcriptomic mapping of human atria
Ion-channel effects in atrial cells	Only ventricular data available	Patch-clamp studies in atrial myocytes
Structural remodeling markers	Non-standard quantification	MRI & biomarker-guided remodeling indices
Distinction from HF benefits	Overlap with hemodynamic effects	AF-focused RCTs controlling for HF status
Combination therapy timing	Limited data	Prospective studies with β-blockers / DPP-4 inhibitors

Unanswered Questions About Atrial-Specific Effects

How do SGLT-2 inhibitors modify atrial remodeling in patients with AF? Such questions are difficult to answer because the proposed mechanisms may not involve the atrium and little data is available on how SGLT-2 inhibition modifies atrial channel proteins or AF-related features. For example, while data indicate that empagliflozin modifies sodium and calcium channel currents in ventricular myocytes, that study did not evaluate the effect on atrial myocytes. Any possible differential effect of SGLT-2 inhibition on atrial or ventricular cells is therefore unknown [36,76]. Nothing is known yet about any possible atrial-selective effects of SGLT-2 inhibition on the cardiac electrophysiological substrate involved in atrial myocyte electrophysiological remodeling, where expression of potassium-translocating enzymes contributes to altered distribution of K+ and Na+ between the intracellular and extracellular compartments. Future studies should examine the direct action of SGLT-2 inhibitors on atrial myocyte ion channels, particularly sodium, potassium, calcium, and calcium-activated non-selective cation channels, which have been implicated in AF remodeling [76]. These new data could clarify whether these agents have specific effects on AF onset and progression.

Design Considerations for Future Trials

One of the main predictors of AF progression and tachyarrhythmia burden is metabolic syndrome, which induces serious metabolic derangements-related consequences on cardiac structure and function-particularly among diabetics [81]. Impaired myocardial glucose and fatty acid oxidation deplete ATP and promote mitochondrial dysfunction, apoptotic signaling, and ferric iron overload, leading to increased ROS production and consequent electrophysiological remodeling. A resultant Na+/Ca2+ overload generates excess diastolic Ca2+ triggering delayed afterdepolarizations, spontaneous activity, and AF initiation [82].

Disruption or dysfunction of organelles (including mitochondria) that liberate ROS during glucose and fatty acid metabolism, via SGLT-2 inhibition, also significantly alleviates AF burden and tachyarrhythmias in these conditions [31]. Moreover, pentose phosphate pathway and hexosamine biosynthesis pathway (HBP) fluxes substantially increase from augmented glucose uptake, altering intracellular sodium and calcium homeostasis and potentially influencing the electrocardiogram of arrhythmic hearts [83]. Therefore, SGLT-2 inhibitors appear to prevent the progression of AF and its tachyarrhythmic burden through improved insulin sensitivity and specific effects on myocardial metabolism, particularly under advanced glycation end products-overload and diabetic conditions [76].

Despite these promising mechanisms and favorable preclinical findings [4], ongoing large-scale clinical trials have yet to determine a direct association between SGLT-2 inhibition and AF. In fact, there is no conclusive evidence on whether SGLT-2 inhibitors exert atrial-specific effects distinct from other CV benefits. Clarification of this mechanistic gap is crucial for linkage between preclinical and clinical studies, substantiating the rationale for ongoing investigation of SGLT-2 inhibitors in AF. Strategies to characterize atrial versus non-atrial effects of SGLT-2 inhibitors could therefore address such preclinical-clinical translational barriers, identify atrial-significant biomarkers and propose experimental platforms or observation in an atrial-dependent model [15]. In patients already prescribed glucose-lowering drugs based on metabolic conditions, concurrent initiation of SGLT-2 inhibitors is emerging as a feasible strategy to evaluate their potential effects on AF incidence and burden [19].

The recent mechanistic insights from cellular models provide compelling biological plausibility for these clinical associations. By activating the PI3K/AKT axis and NRF2 pathway, dapagliflozin mitigates hypertrophy, oxidative stress, and inflammation in both cardiomyocytes and endothelial cells, offering a unifying explanation for its anti-arrhythmic potential [8].

The comprehensive anti-inflammatory and antifibrotic profiles of SGLT-2 inhibitors underscore their potential role as upstream modulators of atrial arrhythmogenesis. The concurrent activation of AMPK and inhibition of NLRP3 inflammasome and NF-κB signaling create a favourable intracellular milieu that limits oxidative injury and maladaptive atrial remodeling. These mechanisms, demonstrated across cardiomyocytes, fibroblasts, endothelial, and immune cells, support a paradigm shift in AF management that integrates metabolic and inflammatory modulation [7]. 

## Conclusions

AF remains a major global health concern owing to its association with increased CV morbidity and mortality. Novel approaches for the prevention of AF are therefore critical. SGLT-2 inhibitors have been associated with a reduced risk of incident AF in patients with type 2 diabetes. Preclinical evidence suggests several potential atrial-specific mechanisms through which efficacy on AF may be mediated. However, clinical data examining the relationship between SGLT-2 inhibitors and AF remain limited. Further studies are required to investigate the impact of SGLT-2 inhibition on atrial remodeling and to clarify the safety and efficacy of SGLT-2 inhibitors on incident, recurrent, and persistent AF.
